# Supplementary material for: Environmentally sensitive hotspots in the methylome of the early human embryo
Source: eLife. 2022 Feb 21;11:e72031. doi: 10.7554/eLife.72031 (PMC8912923; doi:10.7554/eLife.72031)
Supplement: Transparent reporting form [file elife-72031-transrepform1.docx]

***eLife’s* transparent reporting form**

We encourage authors to provide detailed information *within their submission* to facilitate the interpretation and replication of experiments. Authors can upload supporting documentation to indicate the use of appropriate reporting guidelines for health-related research (see [EQUATOR Network](http://www.equator-network.org/%20)), life science research (see the [BioSharing Information Resource](https://biosharing.org/)), or the [ARRIVE guidelines](http://www.plosbiology.org/article/info:doi/10.1371/journal.pbio.1000412) for reporting work involving animal research. Where applicable, authors should refer to any relevant reporting standards documents in this form.

If you have any questions, please consult our Journal Policies and/or contact us: [editorial@elifesciences.org](mailto:editorial@elifesciences.org).

**Sample-size estimation**

- You should state whether an appropriate sample size was computed when the study was being designed
- You should state the statistical method of sample size computation and any required assumptions
- If no explicit power analysis was used, you should describe how you decided what sample (replicate) size (number) to use

Please outline where this information can be found within the submission (e.g., sections or figure legends), or explain why this information doesn’t apply to your submission:

This is a secondary data analysis using data collected from two existing cohort studies. Sample sizes for the two cohorts analysed were therefore determined by the amount of available data. Details are given in the manuscript Results and Methods.

**Replicates**

- You should report how often each experiment was performed
- You should include a definition of biological versus technical replication
- The data obtained should be provided and sufficient information should be provided to indicate the number of independent biological and/or technical replicates
- If you encountered any outliers, you should describe how these were handled
- Criteria for exclusion/inclusion of data should be clearly stated
- High-throughput sequence data should be uploaded before submission, with a private link for reviewers provided (these are available from both GEO and ArrayExpress)

Please outline where this information can be found within the submission (e.g., sections or figure legends), or explain why this information doesn’t apply to your submission:

The study involves comparisons of effects between two independent human cohorts, with an additional longitudinal analysis using data collected at two timepoints for one cohort. Additional steps are taken to test the hypothesis that methylation differences are associated with a periconceptional exposure using additional data from other cohorts. Methylation outliers were removed using Tukey’s method. Further details are provided in the manuscript Results and Methods.

The manuscript contains the following data availability statement:

Illumina 450k methylation array data generated from Gambian 2 year olds from the ENID trial is deposited in GEO (GSE99863). Requests to access and analyse the other Gambian methylation datasets (ENID 5-7yr and EMPHASIS 7-9yr) should be submitted to the corresponding author in the first instance. An application would then need to be made to MRC Unit The Gambia’s Scientific Coordinating Committee and the Joint MRC/Gambia Government Ethics Committee. Sources and locations of other publicly available data used in this analysis are described in Methods.

**Statistical reporting**

- Statistical analysis methods should be described and justified
- Raw data should be presented in figures whenever informative to do so (typically when N per group is less than 10)
- For each experiment, you should identify the statistical tests used, exact values of N, definitions of center, methods of multiple test correction, and dispersion and precision measures (e.g., mean, median, SD, SEM, confidence intervals; and, for the major substantive results, a measure of effect size (e.g., Pearson's r, Cohen's d)
- Report exact p-values wherever possible alongside the summary statistics and 95% confidence intervals. These should be reported for all key questions and not only when the p-value is less than 0.05.

Please outline where this information can be found within the submission (e.g., sections or figure legends), or explain why this information doesn’t apply to your submission:

This information is provided throughout the manuscript in Results and in Figure legends.

(For large datasets, or papers with a very large number of statistical tests, you may upload a single table file with tests, Ns, etc., with reference to sections in the manuscript.)

**Group allocation**

- Indicate how samples were allocated into experimental groups (in the case of clinical studies, please specify allocation to treatment method); if randomization was used, please also state if restricted randomization was applied
- Indicate if masking was used during group allocation, data collection and/or data analysis

Please outline where this information can be found within the submission (e.g., sections or figure legends), or explain why this information doesn’t apply to your submission:

The study is based on a natural experiment (seasonality) which might be described as 'quasi-randomised' by design, in as much as conceptions are randomised throughout the year. However there is the potential for hidden confounding, for example due to epigenetics-related differential survival in early embryos. This is addressed in the Discussion.

**Additional data files (“source data”)**

- We encourage you to upload relevant additional data files, such as numerical data that are represented as a graph in a figure, or as a summary table
- Where provided, these should be in the most useful format, and they can be uploaded as “Source data” files linked to a main figure or table
- Include model definition files including the full list of parameters used
- Include code used for data analysis (e.g., R, MatLab)
- Avoid stating that data files are “available upon request”

Please indicate the figures or tables for which source data files have been provided:

Numerical data represented in the main figures describing season of conception effects are provided in Supplementary Tables.

Data underpinning figures relating to gametic and early embryo methylation data are publicly available, as described in Methods: ‘CpG sets considered in this analysis’. Bespoke code used in the analysis is available at https://zenodo.org/record/580148087.
